# Examining the Direct and Indirect Effects of Postprandial Amino Acid Responses on Markers of Satiety following the Acute Consumption of Lean Beef-Rich Meals in Healthy Women with Overweight

**DOI:** 10.3390/nu16111718

**Published:** 2024-05-31

**Authors:** Morgan L. Braden, Jess A. Gwin, Heather J. Leidy

**Affiliations:** 1 Department of Nutritional Sciences, University of Texas at Austin, Austin, TX 78723, USA; morgan.braden@austin.utexas.edu; 2 Department of Pediatrics, Dell Medical School, University of Texas at Austin, Austin, TX 78723, USA; 3 Military Nutrition Division, U.S. Army Research Institute of Environmental Medicine, Natick, MA 01760, USA; jessica.a.gwin.civ@health.mil

**Keywords:** amino acids, satiety, GLP-1, PYY, mediation analyses

## Abstract

The consumption of protein-rich foods stimulates satiety more than other macronutrient-rich foods; however, the underlying mechanisms-of-action are not well-characterized. The objective of this study was to identify the direct and indirect effects of postprandial amino acid (AA) responses on satiety. Seventeen women (mean ± SEM, age: 33 ± 1 year; BMI: 27.8 ± 0.1 kg/m^2^) consumed a eucaloric, plant-based diet containing two servings of lean beef/day (i.e., 7.5 oz (207 g)) for 7 days. During day 6, the participants completed a 12 h controlled-feeding, clinical testing day including repeated satiety questionnaires and blood sampling to assess pre- and postprandial plasma AAs, PYY, and GLP-1. Regression and mediation analyses were completed to assess AA predictors and hormonal mediators. Total plasma AAs explained 41.1% of the variance in perceived daily fullness (*p* < 0.001), 61.0% in PYY (*p* < 0.001), and 66.1% in GLP-1 (*p* < 0.001) concentrations, respectively. Several individual AAs significantly predicted fluctuations in daily fullness, PYY, and GLP-1. In completing mediation analyses, the effect of plasma leucine on daily fullness was fully mediated by circulating PYY concentrations (indirect effect = B: 0.09 [Boot 95% CI: 0.032, 0.17]) as no leucine-fullness direct effect was observed. No other mediators were identified. Although a number of circulating AAs predict satiety, leucine was found to do so through changes in PYY concentrations in middle-aged women.

## 1. Introduction

Protein-rich foods, consumed as a preload or within a mixed meal, elicit greater postprandial satiety compared to carbohydrate- or fat-rich foods [1,2,3]. These responses are typically accompanied by elevations in several gastrointestinal (GI) hormones, including peptide YY (PYY) and glucagon-like peptide (GLP-1) [2]. Over the past several years, researchers have isolated the secretion of PYY and GLP-1 to the L-cells of the intestinal wall which occurs, in part, by AA stimulation [4,5,6,7]. Despite a consistent, growing body of evidence supporting protein-induced satiety, less is known with respect to the specific AA signals that elicit these responses.

Several AAs (i.e., arginine, glutamine, and the branched-chain AAs—particularly leucine), have been postulated to influence food intake regulation through increased satiety [8,9]. However, most of these data are from infusion studies of single isolated AAs in animal models [8,9]. For example, in rodent models, arginine, glutamine, and leucine infusion studies led to increased secretion of GLP-1 and PYY concentrations, reducing subsequent food intake [10,11,12,13,14,15]. In humans, the relationship between plasma AAs and satiety is generally explored within acute, single-meal studies in which a single protein-rich food or beverage is consumed, and correlational analyses are performed [16,17,18]. Consequently, a novel approach integrating regression and mediation analyses is needed to assess the direct or indirect response of AA predictors and hormonal mediators.

Thus, we sought to extend the current findings to identify which, if any, AAs stimulate satiety following the consumption of a plant-based diet including fresh, lean beef consumed throughout the day. In addition, mediation analyses were performed to determine whether AA-induced satiety occurs through the release of influential gastrointestinal hormones, PYY and GLP-1.

## 2. Materials and Methods

### 2.1. Study Participants

From January 2014 to May 2015, healthy women with overweight were recruited from the Columbia, MO area through advertisements, flyers, and e-mail listservs to participate in the study. Seventeen women signed the consent form and began and completed the study. In general, the women were middle age (33 ± 1 years) and healthy, but overweight (BMI: 27.8 ± 0.1 kg/m^2^). All participants were informed of the study’s purpose, procedures, and risks and signed the consent/assent forms. The study was approved by the University of Missouri Health Sciences Institutional Review Board, and all procedures were followed in accordance with the ethical standards of the Institutional Review Board. The participants received a stipend for completing all study procedures.

### 2.2. Experimental Design

Secondary analyses were performed using a 7-day crossover design study in 17 healthy women with overweight. The purpose of the original study was to examine the effects of red meat consumption as part of a healthy, plant-based dietary pattern on appetite control, satiety, and ad libitum intake compared to a diet void of red meat. On day 6 of each dietary pattern, a tightly controlled testing day was completed consisting of satiety questionnaires and blood sampling performed every 30 min for 12 h for assessments of plasma GLP-1, PYY, and AAs. For this paper, only the red meat dietary pattern was included. Further details of the study design can be found in the following reference [19] and at clinicaltrials.gov as NCT02614729.

### 2.3. Dietary Pattern

For 7 days, the participants were provided with a 2000-calorie plant-based diet containing 2 servings (~7.5 ounces (207 g)) of fresh, lean beef/day for 7 days. The lean beef was in the form of flank and/or top round steak both of which were 95% lean/5% fat. 

The diet included 3 daily meals (i.e., breakfast, lunch, and dinner) and an evening snack. The diet was composed of 16% of energy as protein (76 g protein), 54% of energy as carbohydrates, and 30% of energy as fat. Each meal included ~2 ounces (62 g) of fresh lean beef and the snack included ~1 ounce (27 g) of fresh lean beef. Examples of these meals can be found in the following reference within supplementary data [19].

All study foods were prepared, cooked, and packaged in the metabolic testing facility with each of the ingredients weighed to the nearest tenth of a gram. Participants were instructed to only consume foods provided to them during the intervention period, return all wrappers and uneaten foods to be weighed back, and document any deviations from this protocol such as not consuming all of the meal or eating foods not included in the study meal.

### 2.4. Clinical Testing Day

On day 6 of each pattern, the participants completed the following 12 h testing day: The participants arrived 1 h prior to breakfast, following a 10 h overnight fast, and were taken to a self-contained, comfortable, quiet, and well-lit room to remain there throughout the testing day. The room contained a reclining chair, lamp, laptop (with Wi-Fi), and access to a bathroom. At −60 min, a catheter was inserted into the antecubital vein of the non-dominant arm and kept patent via saline drip. At −30 min, validated computerized questionnaires assessing satiety were completed and a fasting blood draw was performed. Concerning the questionnaires, a 100 mm VAS scale was utilized and the question was worded as “how strong is your feeling of fullness” with anchors of “not at all” to “extremely”. At time +0 min, breakfast was consumed. Throughout the remainder of the day, the same computerized questionnaires were completed, and blood was drawn every 30 min. Lunch was consumed at +240 min, dinner was consumed at +480 min, and an evening snack was consumed at +600 min. At +660 min the catheter was removed, and the participants left the facility.

### 2.5. Repeated Blood Sampling and Plasma Analyses

Blood samples (4 mL/sample; 64 mL/testing day) were collected every 30 min throughout the 12 h testing day. The samples were collected in test tubes containing EDTA (ethylenediaminetetraacetic acid). Protease inhibitors (pefabloc SC and DPP-IV) were added to some of the tubes to reduce protein degradation (for the hormonal analyses) while others did not have the inhibitors (for the amino acid analyses). Within 10 min of collection, the samples were centrifuged at −4 °C for 10 min. The plasma was separated and stored in microcentrifuge tubes at −80 °C for future analysis. Plasma AAs were measured from 13 plasma samples collected across the 12 h testing day. The samples were sent to Pennington Biomedical Research Center—Clinical Chemistry Laboratory and analyzed using High-Performance Liquid Chromatography (HPLC) with o-phthalaldehyde post-column derivatization (Agilent 1000 Series HPLC; Agilent Technologies, Hong Kong, China).

### 2.6. Data and Statistical Analyses

Multivariate regression analyses were performed from the individual time points for plasma AAs, GLP-1, PYY, and fullness responses to determine the plasma AA predictors of satiety. In addition, mediation analyses were performed using PROCESS [20] to examine the direct and indirect effects of specific AAs on satiety through fluctuations in GLP-1 and PYY responses. Note: the AAs selected for the mediation analyses were based on their significance within the regression model. When mediating effects were detected, linear interactions were calculated using PROCESS for the percentiles of the mediator. Bootstrapping was used to generate 95% confidence intervals (CIs) for the interaction parameter calculated in the mediation analysis. Analyses were conducted with the latest version of the Statistical Package for the Social Sciences (SPSS; 29.0; SPSS Inc.; Chicago, IL, USA). *p* < 0.05 was considered statistically significant.

## 3. Results

### 3.1. Amino Acid and Satiety Profiles

**Figure 1:**
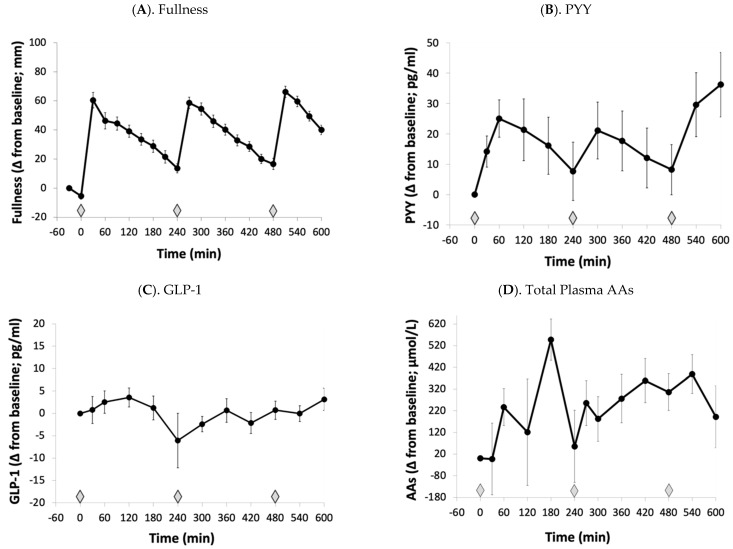
Changes in amino acid and satiety responses across the 12 h testing day in 17 healthy women. The diamond represents the time when the meals were provided. Values are means ± SEMs. GLP-1, glucagon-like peptide-1; PYY, peptide YY; amino acids, AAs; Δ, change.

The pre and postprandial changes in fullness, plasma PYY, GLP-1, and total AAs across the day are shown in Figure 1A–D. Each meal led to immediate increases in fullness and PYY followed by gradual declines until the next meal was provided; however, the postprandial reductions were more blunted with plasma GLP-1 and total AAs compared to fullness and PYY. 

### 3.2. Amino Acid Predictors

**Figure 2:**
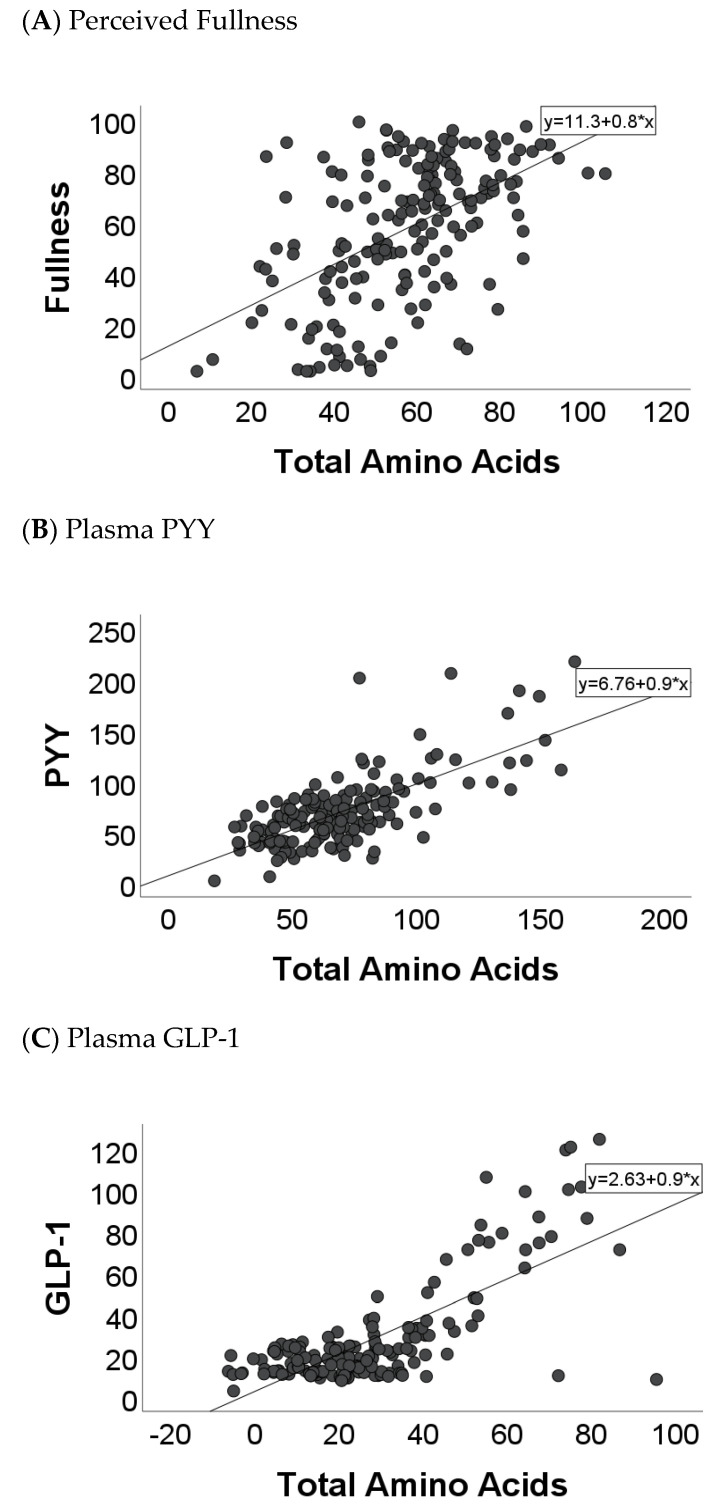
Relationship between satiety markers and total amino acids in 17 healthy women. (**A**) Fullness: r^2^ = 0.41, *p* < 0.001. (**B**) PYY: r^2^ = 0.61, *p* < 0.001. (**C**) GLP-1: r^2^ = 0.66, *p* < 0.001. PYY, peptide YY; GLP-1, glucagon-like peptide-1.

The multivariate regression plots to identify plasma AA predictors of satiety are reported in Figure 2A–C. The full model, including all (total) AAs, explained 41.1% of the variance of perceived daily fullness (*p* < 0.001). When assessing individual AA–satiety associations (Table 1), circulating plasma serine, glycine, alanine, and methionine significantly predicted daily fullness. 

The full model, including all (total) AAs, explained 61.0% of the variance of plasma PYY concentrations (*p* < 0.001). Circulating plasma glutamate, asparagine, serine, histidine, alanine, tyrosine, cystine, phenylalanine, leucine, and lysine significantly predicted circulating PYY concentrations (Table 1). 

Lastly, the full model, including all (total) AAs, explained 66.1% of the variance of plasma GLP-1 concentrations (*p* < 0.001). Circulating plasma glutamate, asparagine, serine, glutamine, glycine, threonine, arginine, alanine, cystine, methionine, isoleucine, and leucine significantly predicted circulating GLP-1 concentrations (Table 1).

### 3.3. Mediation Analyses

**Figure 3:**
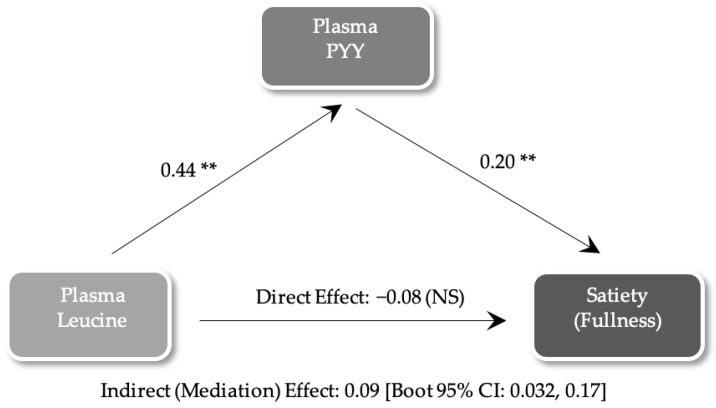
Mediation analyses assessing direct and indirect effects of plasma leucine on satiety; **, *p* < 0.001; NS, not significant.

Mediation analyses were performed on all significant AA predictors to assess direct and indirect effects on satiety. Figure 3 illustrates the only mediation analysis that reached significance. Plasma leucine was significantly associated with plasma PYY concentrations. Plasma PYY concentrations were associated with satiety. However, no direct effect of plasma leucine was observed on satiety. Based on this model, the effects of plasma leucine on satiety were fully mediated by plasma PYY concentrations.

## 4. Discussion

In the current study, we sought to identify which, if any, amino acids stimulate satiety, via select gastrointestinal hormonal secretion, following the consumption of a plant-based diet including fresh, lean beef consumed throughout the day. Although postprandial AA concentrations strongly predicted changes in PYY, GLP-1, and fullness across the day, circulating leucine was the only AA found to elicit satiety through alterations in circulating PYY responses. This study confirms the protein-induced satiety response and provides novel data that identifies leucine as the strongest predictor of satiety through gut hormone activation.

The study of ingestive behavior remains an emerging research target given the impact on diet quality, weight management, overall health, and well-being. One particular dietary strategy that consistently improves ingestive behavior is the daily consumption of increased dietary protein [2]. Substantial evidence from several meta-analyses of intervention trials illustrates that increased dietary protein improves weight management through reductions in daily food intake in combination with greater appetite control and satiety compared to lower protein versions [21,22,23]. In a review by Leidy et al. [2] these hormones appear to be secreted and released to a greater extent following the consumption of higher-protein meals compared to lower-protein meals [2]. While research on protein’s impact on ingestive behavior is abundant, a limited number of studies exist examining the proposed AA signals.

Amino acid secretion has been postulated to influence satiety through the stimulation of GI hormones, PYY, and GLP-1. Similar to the findings in our current study, several human studies report associations between plasma AA concentrations and markers of satiety following the consumption of whole protein or AA-enriched sources [17,18,24,25]. For example, Rigamonnti et al. [17] assessed the satiety effects of AAs following the consumption of whey protein (45 g) compared to a carbohydrate control. A number of postprandial circulating AAs, including leucine, were negatively correlated with hunger and positively correlated with fullness and GLP-1 concentrations. However, in contradiction to our current findings, no AA-PYY associations were observed. Yanni et al. [25] provided subjects with arginine or branched-chain AA-enriched biscuits to measure plasma AA concentrations and markers of satiety. While they too found significant increases in plasma AAs, subjective appetite and satiety measurements, and postprandial GLP-1 response following both enriched bars, plasma PYY levels were unchanged. In a study by Uhe et al. [18] AA–satiety associations were identified following the consumption of 2 oz portions of beef, chicken, or fish whole-food protein sources. Postprandial taurine and methionine concentrations were associated with satiety following the fish meal, but not with beef or chicken. These findings contradict the associations observed in the current study that incorporated fresh, lean beef within a mixed meal as part of a healthy dietary pattern. Uhe et al. provided meals consisting of only a piece of grilled meat, which suggests that the differences in study findings may occur as a result of the inclusion of beef within a meal–matrix paradigm in our current study.

A proposed AA nutrient signal for protein-induced satiety is the essential BCAA leucine with PYY mediating this response [4,6,26]. Through in vitro studies, leucine was detected to bind to receptors, T1R1/T1R1 heterodimers, CaSR, and GPR93, in the ileum to stimulate the release of PYY [4,5,6,7]. PYY either binds to the Y2 receptor of the vagus nerve or is secreted into circulating blood to exert its effects centrally—either through stimulation of the nucleus tractus solitarius in the brain stem or via stimulation of the NPY/AgRP neurons of the arcuate nucleus of the hypothalamus to influence ingestive behavior. Given this proposed pathway, Bolster et al. [24], conducted an acute crossover trial to assess the consumption of leucine-enriched bars on postprandial satiety and plasma AA levels. The consumption of leucine-enriched bars increased satiety more so than the consumption of bars without leucine. In addition, leucine was found to be associated with PYY concentrations. This gut–brain satiety axis is supported by the postprandial leucine-stimulated satiety response, mediated by plasma PYY, observed in our current study.

Recent nutrition recommendations suggest substituting animal-based protein sources with plant-based ones in an effort to encourage individuals to shift toward plant-based dietary patterns [27]. However, the protein quality differences between these protein sources have been questioned. Animal-based proteins have a greater essential AA profile, specifically increased leucine, and digestibility compared to plant-based sources [28,29]. As supported in the current study, the consumption of fresh, lean beef led to AA-induced satiety, which was attributed to leucine absorption. The removal of higher-quality animal protein sources from the diet may diminish this significant AA–satiety response and negatively influence eating behavior over time. Further research is needed to support the role of dietary protein sources within meal matrices on AA stimulation of satiety responses.

### Limitations

Although our design extends the existing evidence by assessing AA predictors, potential hormonal mediators, and satiety across the day, some limitations should be noted. We included a repeated, mixed-meal design in which whole protein foods were consumed along with carbohydrates and fats. As shown in a number of reviews [30,31,32], other nutrients can either directly elicit satiety (e.g., fiber, calcium, iron, and B12), or indirectly do so through altering protein digestibility. Despite these nutrient confounders, postprandial AAs in our current study accounted for a large variability in the daily satiety response [30]. Additionally, this design may also be considered a strength as most individuals consume their protein within mixed meals. Lastly, while a number of circulating AAs predicted satiety, only leucine was shown to stimulate satiety via PYY concentrations. These findings are novel and meaningful but do not negate the potential role of other AAs to elicit satiety via additional mechanisms that were not explored in this study.

## 5. Conclusions

Although circulating postprandial AAs strongly predict daily satiety, leucine was found to elicit this response via gut-derived secretion of PYY. These data confirm AA-induced satiety responses and support the consumption of animal-source foods, rich in essential AAs, like leucine, within a healthy dietary pattern in healthy women with overweight.

## Figures and Tables

**Table 1:** Multivariate regression analyses assessing pre- and postprandial plasma AAs as predictors of satiety. * *p* < 0.05.

Fullness	Plasma AA	*B*	*SE B*	β	*t*	*p*
	glutamate	−0.81	0.150	−0.51	−0.542	0.589
	asparagine	0.838	0.245	0.187	1.565	0.120
	serine	0.520	0.175	0.416	2.969	0.003 *
	glutamine	−0.008	0.041	−0.022	−0.205	0.838
	histidine	−0.340	0.317	−0.152	−1.071	0.286
	glycine	−0.119	0.041	−0.373	−2.906	0.004 *
	threonine	0.140	0.123	−0.152	1.134	0.259
	citrulline	0.165	0.578	0.035	0.285	0.776
	arginine	−0.148	0.187	−0.133	0.792	0.429
	alanine	0.167	0.033	0.504	5.001	<0.001 *
	tyrosine	0.266	0.416	0.082	0.639	0.524
	cystine	−0.695	0.429	−0.330	−1.619	0.107
	valine	0.078	0.222	0.086	0.352	0.726
	methionine	−2.716	0.917	−0.458	−2.963	0.004 *
	phenylalanine	1.216	0.716	0.367	1.699	0.091
	isoleucine	0.437	0.433	0.192	1.011	0.314
	leucine	−0.140	0.443	−0.096	−0.316	0.752
	lysine	−0.146	0.129	−0.195	−1.132	0.259
R^2^ = 0.411 F (18,159) = 6.170						
PYY	Plasma AA	*B*	*SE B*	β	*t*	*p*
	glutamate	0.748	23.340	0.370	4.418	0.000 *
	asparagine	0.771	0.169	0.297	2.906	0.004 *
	serine	−0.632	0.265	−0.393	−3.233	0.002 *
	glutamine	0.011	0.195	0.023	0.250	0.803
	histidine	0.874	0.046	0.307	2.527	0.013 *
	glycine	0.075	0.346	0.183	1.669	0.097
	threonine	0.105	0.045	0.094	0.776	0.439
	citrulline	0.405	0.135	0.067	0.640	0.523
	arginine	0.170	0.633	0.117	0.808	0.420
	alanine	0.192	0.210	0.453	5.348	0.000 *
	tyrosine	−1.521	0.036	−0.362	−3.276	0.001 *
	cystine	−2.479	0.464	−0.916	−5.141	0.000 *
	valine	−0.475	0.482	−0.397	−1.956	0.052
	methionine	−1.347	0.243	−0.177	−1.321	0.189
	phenylalanine	3.933	1.020	0.916	4.806	0.000 *
	isoleucine	−0.883	0.818	−0.295	−1.808	0.073
	leucine	1.230	0.489	0.639	2.565	0.011 *
	lysine	−0.318	0.480	−0.327	−2.223	0.028 *
R^2^ = 0.610 F (18,145) = 12.578						
GLP-1	Plasma AA	*B*	*SE B*	β	*t*	*p*
	glutamate	0.453	0.111	0.319	4.069	0.000 *
	asparagine	−0.792	0.175	−0.431	−4.514	0.000 *
	serine	−0.460	0.129	−0.407	−3.579	0.000 *
	glutamine	0.184	0.030	0.533	6.139	0.000 *
	histidine	−0.317	0.227	−0.157	−1.394	0.166
	glycine	0.124	0.030	0.429	4.180	0.000 *
	threonine	0.409	0.089	0.523	4.616	0.000 *
	citrulline	−0.742	0.416	−0.174	−1.786	0.076
	arginine	−0.414	0.138	−0.407	−3.003	0.003 *
	alanine	0.061	0.024	0.205	2.587	0.011 *
	tyrosine	0.425	0.305	0.144	1.395	0.165
	cystine	−0.884	0.317	−0.463	−2.788	0.006 *
	valine	0.285	0.160	0.340	1.787	0.076
	methionine	1.789	0.670	0.335	2.670	0.008 *
	phenylalanine	−0.191	0.540	−0.063	−0.353	0.724
	isoleucine	0.647	0.322	0.307	2.010	0.046
	leucine	−0.815	0.316	−0.602	−2.584	0.011 *
	lysine	0.051	0.095	0.074	0.539	0.591
R^2^ = 0.661 F (18,144) = 15.630						

## Data Availability

The data presented in this study are available on request from the corresponding author due to privacy reasons.
